# Community biocultural mapping reveals historical occupation and enables defense of African rainforests

**DOI:** 10.1007/s13280-025-02334-2

**Published:** 2026-02-20

**Authors:** Ibola Dja Bana Ba Massaha, Serge Ekazama Koto, Gretchen M. Walters, Honorine Asatsi Mabo, Fulbert Makala, Paulin Ndanga Azeon, Jean Mabo, Philippe N. Mandoumilele, Jean P. Hendje, Dieu-Donné Baza Djia, HB Betotobeya, Modeste Ndongoabendje, Ernest Maïdji, Story Maloumambomba, Djiese Koumokoukou, Germain Kotomoukaye, Fulgence Mbengoy, Boris Bobouagno, Garance Bopounda, Dieu-Donné Ngouba, Yanick Akouboua, Mathurien Haya, Steeven Mabe Bleck, Gervais Djabouemi, Aimé J. Bobeloubouangoy, Vincent Malingui, Davy Ikakaboua, Didier T. Louma, Paul Mbouya, Guy R. Imbembi, Felix Eboulou, Patrick Ipengongoy, George Moulingui, Hines Mabika, Julia Walker, Médard Mamouaka Bayadi, Simon Cheseaux, Alex Ebang Mbélé, Graden Z. L. Froese

**Affiliations:** 1 Community of Massaha, Massaha, Gabon; 2 Nsombou Abalghe-Dzal Association, Makokou, Gabon; 3 Faculty of Geosciences and the Environment, Institute of Geography and Sustainability, University of Lausanne, Géopolis, Quartier Mouline, Lausanne, Switzerland; 4 Institute for the History of Medicine, Universität Bern, Bern, Switzerland; 5 Institut de Recherche en Ecologie Tropicale, Libreville, Gabon

**Keywords:** Big data, Biocultural, Central Africa, Community, Conservation mapping methods, Intact forest

## Abstract

Global conservation maps often overlook community lands, impacting decision-making and reducing local agency. We focus on the high-profile and regionally important case of Massaha’s ancestral territory (Ibola Dja Bana Ba Massaha). In Gabon, the forest is largely gazetted into logging concessions and described as an Eden-like wilderness despite long-term human presence. We compare Massaha’s biocultural maps to global, colonial, and post-colonial maps, in a community peer review process. While these maps show Ibola Dja Bana Ba Massaha as devoid of logging and settlement, Massaha’s maps show long-term settlement, sacred areas and industrial logging. Massaha used their maps to request the government to protect their forest from logging and update the forestry code to enable community conserved areas, prompting a national debate on the legal recognition of “territories of life”, while revitalising ancestral practices. We show that “big data” conservation mapping must account for biocultural knowledge if it is  to be effective in guiding conservation decisions.

**Supplementary Information:**

The online version contains supplementary material available at 10.1007/s13280-025-02334-2.

## Introduction

Conservation policy often uses global mapping to prioritise areas of intervention (Myers et al. 2000). However, global maps may fail to capture long-term land occupation, thus potentially contributing to the exclusion of community land governance and management in conservation policy. In response, Wyborn and Evans (Wyborn and Evans 2021) encourage conservation actors to “break free” from global maps and instead re-engage with and embrace empirical studies of local and regional contexts.

Communities have been living in, using and caring for most our planet’s ecosystems for over 12 000 years (Ellis et al. 2021), leaving a mark on its biodiversity (Morin-Rivat et al. 2017). Indigenous Peoples and local communities govern vast areas of the globe, around 40% of “ecologically intact landscapes” (Garnett et al. 2018), and have an increased likelihood of protecting their resources (Sze et al. 2022). In-depth studies show how Indigenous Peoples and local communities contribute to conservation through the traditional resource management of their territories from Oceania to Africa, Asia, Europe, and the Americas (Zanjani et al. 2023). We question what maps are available to guide the understanding of community long-term occupation and how these contribute to community-led conservation.

Maps have the potential to inform conservation action and understand past landuse. However, reading the history of these landscapes is challenging (Fairhead and Leach 1996), and related mapping exercises may significantly misrepresent who manages different geographic areas and through what means (Harris and Hazen 2005). Maps can also have unintended consequences such as misidentifying community lands and causing conflict over resource use and access (Harley 1988). This may be particularly the case for areas where global maps influence locally-made decisions concerning natural resources. In Central Africa, the world’s second largest rainforest after the Amazon (Hubau et al. 2020), global and regional maps often characterise these forests with a near absence of human influence; these same maps influence local forestry management and conservation decisions.

We focus on a highly influential global map, Intact Forest Landscapes (IFLs) and a global deforestation tracking tool, Global Forest Watch’s Integrated Deforestation Alerts. IFLs are defined as “a seamless mosaic of forest and naturally treeless ecosystems with no remotely detected signs of human activity and a minimum area of 500 km^2^” (Potapov et al. 2017). While IFLs and similar global maps can show the absence of satellite-observable industrial activity, they do not factor in what happens below the canopy, such as hunting pressure (Haurez et al. 2017). IFLs also often become quickly outdated due to rapid logging and road expansion (Kleinschroth et al. 2019). Global Forest Watch’s Integrated Deforestation Alerts,^1^ which give daily 10 × 10 m resolution data on forest or tree cover disturbances, can help fill this gap by providing timely information to track and address logging.

Furthermore, IFLs are not able to account for the presence of community lands and sacred areas, in part because these maps are devoid of historic and biocultural information. Although there is a recognition that Indigenous peoples and local communities rely on IFLs without necessarily negatively impacting them (Watson et al. 2018), these global analyses inherently, even if unintentionally, suggest that these forests are not heavily used and are largely uninhabited. The approach of High Conservation Value Forest assessment promoted by the FSC could fill this gap, but only in forests which are subject to certification, and so is not applicable to most forestry concessions (Areendran et al. 2020; Matias et al. 2024).

How can global maps be redefined at local scales to inform conservation policy and incorporate possible long-term occupation? Biocultural approaches to conservation are pathways to achieving just and effective conservation (Gavin et al. 2018). We suggest that a local approach, using community-led biocultural mapping of their territory, can rewrite global maps and reveal the role communities play in conservation.

In Gabon, as in much of Central Africa, human settlement patterns over time were mobile. Gabon has been inhabited at least since 620 000 years (Braucher et al. 2022). Much of the country, including those who lived in the northeastern region of this study, had a communal society with a lifeway based on gathering, hunting, and fishing (collectively defined in the Kota language as *nsombou*) that drove them to regularly and seasonally relocate their homes, based on the availability of natural resources (Deschamps 1962).

We present the critical case study (Flyvbjerg 2011) of the Kota community of Massaha’s ancestral territory in Gabon to illustrate why local biocultural mapping is important for just conservation and how it grounds—and contrasts with—global maps in the context of "big data". Gabon is the ideal country for understanding how local people can demonstrate long-term occupation of their forests when faced with global extraction processes such as logging: it is one of the world’s 11 high-forest, low-deforestation countries and has the highest proportional forest cover in Africa (Takougoum Sagang et al. 2024) and contains extensive logging concessions (Legault and Cochrane 2021). Many of these concessions are guided by Forestry Stewardship Council certification, which is becoming required in Gabon (Brouwer 2021), and IFL zoning suggests community engagement policies (Forestry Stewardship Council 2020). Gabon has a low rural human population density and a government committed to area-based conservation (Doumenge et al. 2021) and is often presented as an Eden or wilderness (Quammen 2003; Cinnamon 2003) despite a sustained history of Indigenous peoples (Olivero et al. 2016) and forest-dependent Bantu-speaking communities (Oslisly 2001; Mabika Ognandzi 2019).^.^Further, Massaha represents a unique opportunity to probe the relationship between a local community’s biocultural approach versus global landuse mapping because, marking an unprecedented move in Gabon, the community mapped part of their ancestral territory and formally requested the government to remove it from a logging concession and reclassify it as a community conserved area with sustainable use, to be called “Ibola Dja Bana Ba Massaha” (“the reserve of all Massaha’s children”; see “Massaha’s fight to save their ancestral territory” section). This case has become a catalyst for legal change towards recognising community conserved areas (Evine-Binet 2023).

In this paper we, a group of Massaha community members, a local NGO, and researchers, present our methods used to understand long-term forest occupation, which contrasts with colonial, post-colonial and global landuse maps that have consistently presented this area as largely uninhabited. We do so through the principle that sincere partnership of communities and researchers can deepen the understanding of biocultural heritage and relationships to land (Molnár et al. 2023). We compare colonial, post-colonial, and global landuse maps with Massaha’s biocultural mapping. We ask: how do colonial, post-colonial, and global landuse maps represent community land occupation? How can community biocultural mapping demonstrate long-term occupation of ancestral territory? And how does it contribute to its conservation?

## Materials and methods

### Study site: Massaha and the Ogooué-Ivindo

The Ogooué-Ivindo province is the largest province of Gabon with ~ 4.6 Mha, and one of the richest in resources yet least developed. Gabon’s colonial legacy from France left its forests managed largely as large foreign-owned concessions (Legault and Cochrane 2021) and colonial authorities repressed local people, whose labour was targeted to extract timber resources for the colony (Tsanga et al. 2022). From 1889 onward, the colonial exploitation of timber brought a great change in both the Gabonese economy and people’s relation to forests as concessionary companies could manage thousands of hectares of lands as well as the people living in them (Coquery-Vidrovitch 1972). However, some forests remained largely unexploited until recent decades, given Gabon’s zonal approach to forestry which reserved the interior forests to be the last to be exploited (these being difficult to access, furthest from the coast, and lacking the Okoumé tree, a high-value tree used to make plywood); the interior forests began to be exploited for diverse species of hardwood in the 1980s with the construction of the railroad (Pourtier 1989). The forest of Massaha is in this latter zone; logging began in earnest in the area in 2000s. Furthermore, a Gabon-wide colonial resettlement policy (*regroupement*) forced many communities to abandon their villages and surrounding territories and move to the roadside, leaving much of the forest to appear abandoned (Sautter 1966; Cinnamon 2003). The appearance of vacant forest enabled the creation of 13 national parks in 2003, without displacing people by largely reducing access (Walters and Wardell 2024). Nonetheless, present-day villages in Gabon maintain strong ties with their territories from which they were resettled (Walters et al. 2015).

After a period of migration (Perrois 1970), the modern-day community of Massaha was formed through two *regroupements* (grouping of several villages for administrative purposes)*,* the first of three villages in 1951 with two more being added in 1970. Massaha is located 56 km to the northeast from Makokou, the provincial capital of the Ogooué-Ivindo. The community’s ~ 70 households are distributed as two parallel rows of houses on either side of the unpaved national road. People live primarily through agriculture, gathering, hunting, fishing, intermittent employment in a nearby mill and in the forest beyond their territory (e.g. small-scale gold mining, prospecting for logging companies), or city-work in Makokou and beyond. North of the road, Massaha’s territory has been leased by the state to create a community forest of ~ 6600 ha, which they began logging in 2017, with revenue attributed to community development projects. South of the road is where this study takes place.

### Massaha’s fight to save their ancestral territory

Massaha’s ancestral forests south of the national road are part of a forestry concession allocated to Transport Bois Négoce International (TBNI), a Chinese logging company known for illegal practices (EIA 2019). Logging roads started being built into the area in 2020. In an unprecedented case in Gabon, in an attempt to save their ancestral forests from logging, the Massaha community mobilised and formally applied, using legal processes outlined in the Gabonese forestry code, to remove their ancestral lands from TBNI’s concession and to classify the same area as a community conserved area with sustainable use. First in a 2020 General Assembly, the community decided to address a dossier to the *Ministère des Eaux et Forêts¸* the province’s Governor and the TBNI, and subsequently in tens of letters and meetings within the village and with the government in the following years up to the time of publication. The people of Massaha wrote in their first letter that “logging in this area will destroy all the foundations of our village. We do not want to be a village with no roots and no history” and later proposed their desired conserved area be called “*Ibola Dja Bana Ba Massaha*” (which means “the reserve of all Massaha’s children”).

### Community biocultural maps

In 2021, we used a Participatory Geographic Information System (PGIS) that we created in R Shiny (originally to explore community hunting territories, and later updated to map ancestral territories in general (Froese et al. 2022)) in a peer review process by community members (Liboiron et al. 2018). In a community-wide meeting, a map was projected onto the wall with background data, such as roads and rivers, as well as the community’s hunting tracks. On to this map, community members directly “drew” (by directing the facilitator’s use of the computer) their perceived/estimated boundaries of their ancestral territories, as well as the locations of ancestral villages and other biocultural sites within. Community members and the facilitator then walked in the forest to geolocate with GPS units as many of these perceived/estimated points as possible. Later, we held oral mapping sessions in which elders listed and detailed biocultural sites that may have been missed in that first mapping meeting. As Massaha’s movement gained momentum and the government requested an updated map in 2022, many community members spent a week in the forest, followed by five separate day walks, to refine their ancestral boundaries with neighbouring villages and geolocate any ancestral villages, sacred sites, and baïs (swampy forest clearings) still missing from their map. These new data were used to update Massaha’s map, which was validated in a community-wide meeting before being provided to the government.

At that point the map resembled the one displayed in Fig. 2. Massaha continued, and continues, to update information as they conduct monitoring of biocultural diversity and patrol logging activity. Iterations of these maps are shared with the government and other parties depending on the specific issue at hand to be communicated. Massaha monitors these sites over time (as well as illegal logging practices), conducts biodiversity and carbon research in some of them, and archives their oral history from them for future generations.

### Colonial maps

We obtained nine colonial maps of the greater Makokou area from colleagues as well as from archival work in the Archives National d’Outre-Mer (Aix-en-Provence, France) in 2021 and 2022 with maps dating from 1897, 1911 (two maps), 1929, 1933, 1946, 1950s, 1966, and 2022. The colonial maps were photographed by the archives at a high resolution.

We used QGIS 3.10 to georeference these maps. We set the Coordinate Referencing System (CRS) to WGS 1984 (EPSG: 4326) and imported a Google Satellite Hybrid Basemap from the QGIS toolkit to use as the basemap that the colonial maps would be georeferenced to. We used the georeferencer tool in QGIS to complete georeferencing for each colonial map. Topological features that were unlikely to have changed between the years of the map and current day were located and used as control points, as suggested by typical georeferencing methods (Hackeloeer et al. 2014). These included features such as rivers and roads. We placed a ground control point (GCP) on the historical map feature and then at the corresponding location on the basemap on QGIS (Environmental Systems Research Institute 2023); we repeated this process for each feature and kept GCPs with the minimum possible error (residuals) between the historical map and the current basemap. We generated a minimum of four GCPs before applying either a linear or a first order polynomial transformation to the colonial map to georeference it (Hackeloeer et al. 2014; Environmental Systems Research Institute 2023). Depending on which yielded a more accurate transformation, we used either a linear or first order polynomial transformation. We often produced both transformation types to decide which method obtained a more accurate output.

We also created historical village layers in QGIS 3.26. Once a colonial map was georeferenced, we made a village layer with the CRS WGS 1984 (EPSG: 4326). We created a new point shapefile to represent each village layer. If there were multiple points on the map that represented villages, we gave each documented point on the historical map a point in shapefile format. If there was doubt on whether a dot represented a village, we first consulted the map legend, then if there were no other obvious markings (such as those representing schools or churches) we included the dot as a village location.

### Global landuse maps

We obtained the 2020 Intact Forest Landscape (IFL) shapefile layer for Gabon from the Intact Forest Landscapes (intactforests.org) initiative and imported it into RStudio in order to compare the IFL with the historic and current village layers. We downloaded Global Forest Watch’s Integrated Deforestation Alerts (from August 16th, 2020, the first available data after Massaha submitted its request, to February 15th, 2025, the most recently available data for  analysis conducted on March 4th, 2025) and visually compared them with data collected by community patrols of logging. We further obtained shapefiles of Gabonese villages, logging concessions, national parks, major rivers, and national roads commonly used by government, civil society, and researchers. We did not include logging roads, as available government data and satellite products vastly underestimate the extent of logging roads in our study area (Kleinschroth et al. 2019). We created summary statistics of IFLs using spatial geometry in Rstudio (package sf version1.0–15).

### FPIC process of data sharing and use

In order to bring our datasets together, in 2023, the researchers, with the support of NADA, presented their colonial map findings to members of Massaha during a village meeting, provided copies of their maps, and explained the project presented in this paper. Community members agreed through a Free, Prior, Informed Consent (FPIC) process to share their data for the purpose of this project. Confidence with the community was possible given their existing partnership with researchers as members of NADA  and their previous experience in co-authoring research. In a community meeting in 2024, community members agreed that the first author of the paper should be the name of their territory in order to respect the role of the land and water in conserving their territory; other instances of land-based authorship have been used in similar situations (Country et al. 2016; M’sit No’kmaq et al. 2021; Mau Forest et al. 2025).

## Results

### Community biocultural maps

**Fig. 1:**
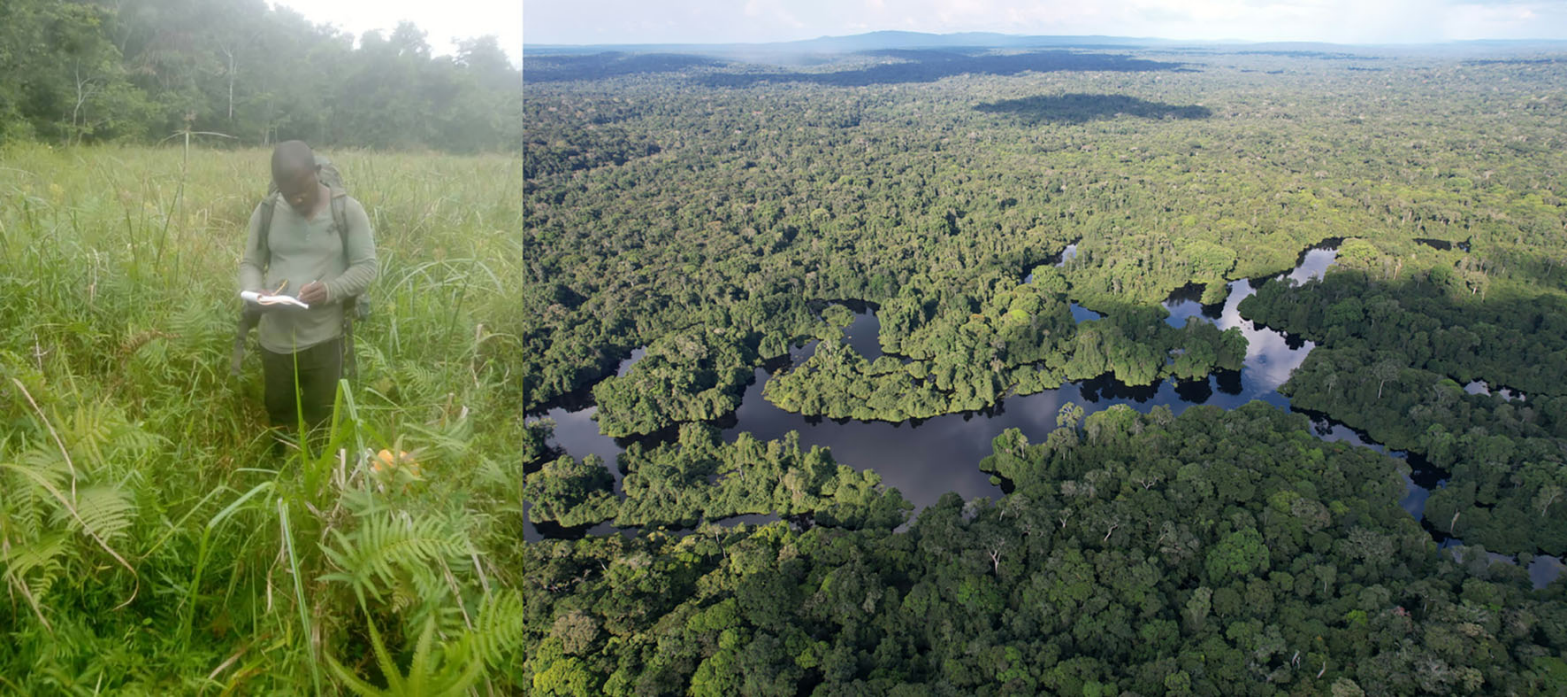
Left: Paraecologist and author Serge Ekazama Koto  maps a previously undocumented baï, a swampy forest clearing important to biodiversity. Right: aerial view of Ibola Dja Bana Ba Massaha showing the *issuaka* (mature forest) south of the Liboumba (Abombwé) river. Near the river are many ancestral villages and sacred sites unmarked by colonial and global maps; the forest in the background was classified as within an Intact Forest Landscape (IFL) in 2020 but has since been logged. Photos by Guy Rodger Imbembi and Walter Mbamy

The community of Massaha, with the technical facilitation of a local NGO, NADA, mapped their ancestral territory through an approach combining oral histories of ancestral knowledge, a novel participatory geographic information system (PGIS), ground-truthed geographic information system (GIS) data collection, and community data review (Fig. 1).

**Fig. 2:**
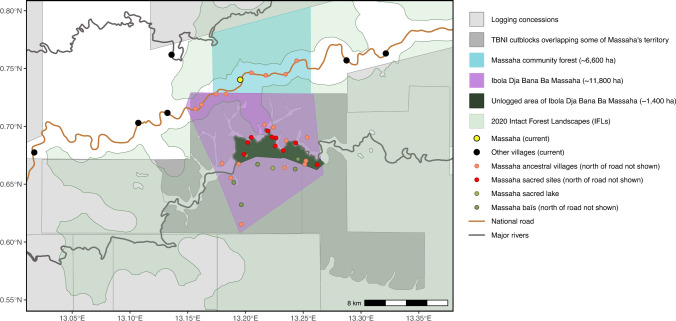
Community biocultural mapping of Ibola Dja Bana Ba Massaha: community-collected data on biocultural sites and territorial limits (north of the national road are not shown), presented along with available data on logging concessions, roads, rivers, and current villages. Also presented, for the purpose of this study, are 2020 Intact Forest Landscapes (IFLs)

Beyond mapping the overall ~ 11 800 ha of Ibola Dja Bana Ba Massaha, the community mapped features within it (most of which are concentrated not around the current village, but several km to the south nearby the Liboumba (Abombwé) river) as well as the impact of logging (Fig. 2). These features include 15 ancestral villages (14 destroyed/impacted by logging and one intact), 1 sacred lake (impacted by logging), 10 sacred sites (two destroyed/impacted by logging and eight intact) and six swampy forest clearings also called baïs (four destroyed/impacted by logging, two intact). The Massaha community has occupied the area from at least the 1800s to present.

### Colonial maps

**Fig. 3:**
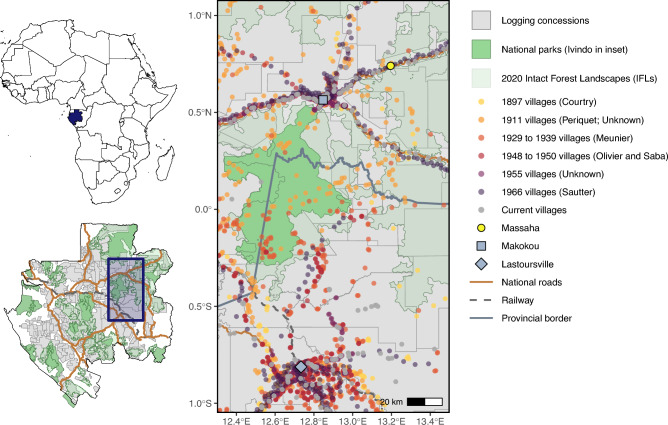
Composite map showing the overall settlement patterns over time as mapped by colonial and post-colonial (after 1960) mapmakers. Shown in relation to current logging concessions, national parks, Intact Forest Landscapes (IFLs), villages and towns, and national roads and the railway

When viewed together at a landscape scale, maps from 1897 to present show a broad settlement pattern; more numerous villages are present on maps far to the south of Massaha (Lastoursville area) than nearby (Makokou area; Fig. 3). By 1966, the maps show the villages aligning along roads as a result of a Gabon-wide colonial resettlement policy (*regroupement*) enacted from the early 20th century and continued by the Gabonese government after independence in 1960 until the 1970s, forcing many communities to abandon living in their villages and move to the roadside, leaving much of the forest to appear no longer inhabited (Sautter 1966; Cinnamon 2003; Walters et al. 2025).

**Fig. 4:**
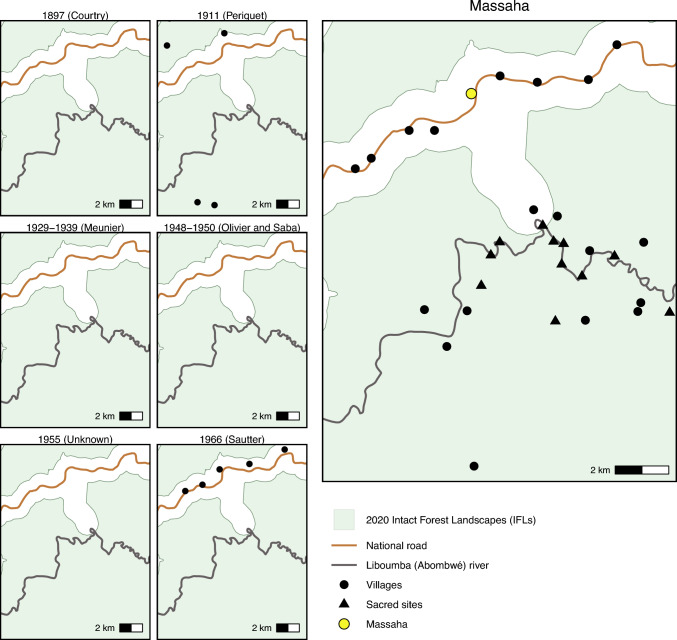
A comparison of Massaha’s biocultural map (right), with the colonial and post-colonial maps (left). Massaha’s biocultural sites north of the national road are not shown. Multiple colonial maps by the same author over several years are grouped together

When we compare the community maps with the colonial and administrative maps (Fig. 4), we find that only one of four mapping initiatives before 1951 refers to any of Massaha’s ancestral villages, and this map only shows two villages. By contrast, Massaha’s mapping of the area south of the road shows 15 ancestral villages: an increase of 88%. None of the 11 sacred sites mapped by Massaha are shown on the colonial maps. A 1955 map does not show any of Massaha’s more recent roadside ancestral villages, while shortly after Independence, in 1966 a map shows five. Colonial and post-colonial maps are thus not able to account for historic village settlement and probably severely underestimate historic human settlement over a wider area.

### Global landuse maps

Gabon’s national territory of ~ 26.7 Mha has a forest cover of nearly 88%: ~ 11% of the country is classified as national parks, and as of 2019, ~ 58% as logging concessions (Conseil national climat Gabonais 2020). In 2020, IFLs covered ~ 29% of Gabon^8^. Ibola Dja Bana Ba Massaha's ~ 11 800 ha was classified as ~ 86% IFL in 2020, a status which would typically help to protect some of the area under FSC policy. Yet, selective logging near the village had happened in the mid-2000s prior to the arrival of the TBNI logging company (which is currently uncertified), and in 2020, the only unlogged area within Ibola Dja Bana Ba Massaha was the ~ 5300 ha south of the Liboumba (Abombwé) river. In 2023, TBNI delimited an area of ~ 1400 ha (~ 26%) of this previously unlogged forest that they had agreed in writing not to log.

**Fig. 5:**
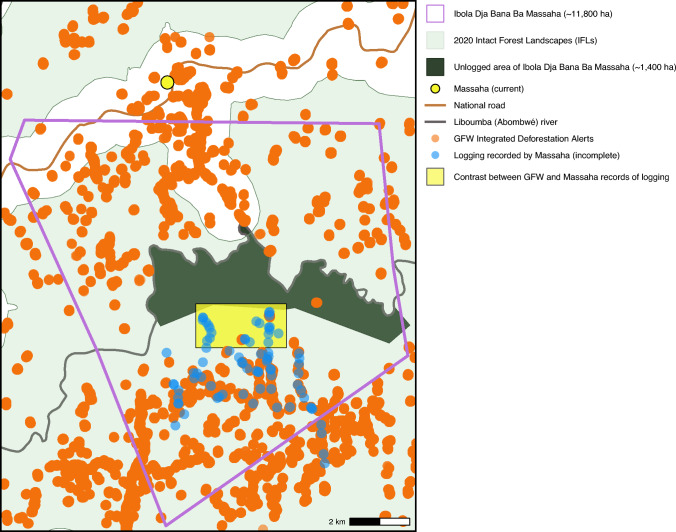
Logging mapped by Massaha (a July 2023 community patrol), some of which was not detected by Global Forest Watch’s Integrated Deforestation Alerts (from August 16th, 2020, the first available data after Massaha submitted its request, to February 15th, 2025, the most recently available data as of analysis conducted on March 4th, 2025)

In order to understand how community-led biocultural mapping can contribute to conservation, we compare the Global Forest Watch’s Integrated Deforestation Alerts with Massaha’s mapping of logging in their territory, which they strategically undertook at key times during their struggle in order to alert partners and the government about illegal logging practices^2^ or incursion into areas that had agreed upon to be spared, in several instances saving these areas. For example, a community patrol in July 2023 showed logging in areas with no Integrated Deforestation Alerts (Fig. 5). The satellite data indicates almost no logging for a ~ 2 km band south of the truly unlogged area, essentially doubling the amount of area that one would assume to be unlogged.

## Discussion

### Causes and consequences of colonial mapping

Villages in Central Africa were previously mobile in response to many factors: in the past villages were often moved voluntarily due to natural resource conflict, inter-ethnic war, and sorcery (Soret 1973). Migration of ethnic groups such as the Kota of Massaha, also led to moving village sites (Perrois 1970). But such moves were mainly initiated by people themselves. During the colonial era, regroupement policy along national roads contributed to village displacement, but this time, not by the will of the people, but by an obligation of the colonial power, as it was the case for the Kota communities gathered at Massaha. In this paper, colonial maps were hypothesised to help understand broad settlement patterns and snapshots of village settlement over time. At a larger scale, maps show that villages were scattered throughout the forest of the wider study site. However, even at this larger scale, large areas of the forest appear to be uninhabited, despite extensive landuse for hunting, gathering, and customary practices (e.g. sacred areas) (Walters et al. 2015). Resettlement policy in Gabon forcibly resettled people and villages over a period of more than 50 years. In some cases, villages were repeatedly displaced, even over small distances, and at the same time, many villages resisted resettlement and repeatedly returned to their ancestral territories (Sautter 1966). This makes colonial maps inadequate to assess forest occupation over time: the colonial mapmakers did not necessarily map villages, and did not repeatedly visit and map the same sites, unless there was a colonial interest in commerce, labour, or controlling the population. Strategic or potentially strategic areas, in terms of rebellion, military intervention or economic potential often justified a higher level of village documentation (Brunschwig 1972). For example, the Lastoursville area (Fig. 3), may be well documented because it was the site of one of the great Gabonese uprisings against French Colonists, the War of Wongo of 1928–1929 (Métégué N’Nah 2006). We suggest that the higher density of villages in the colonial maps around the Lastoursville area was related to colonial efforts to track settlement and control uprisings. Although oral histories of the greater Makokou area also relate wars, as well as colonial resistance (Perrois 1970), these villages remain largely undocumented on the colonial maps we present in this study.

The colonial administration was interested in commercial development, labour, and taxes (Coquery-Vidrovitch 1972) and so colonial maps provide largely a view of extraction, with specific maps (e.g. Perriquet 1911, Fig. 3) made for creating a railroad (where the interest was more in topography than human settlement). The content of colonial maps changes given the context of the former colony; in Liberia, for example, colonial maps did not document villages as the mapmakers did not think it was worthwhile (Rossinelli 2022). In rare cases, colonial maps were made in collaboration with local people (Palsky 2013). Nonetheless, in many cases, it is difficult to understand the objective of a colonial map since the maps were often disassociated from related reports and even separated across different archives (Olive 2019). So, although our series of maps at a large scale shows former villages scattered throughout the forest, they do not represent what has happened over time at a local scale, and likely severely underestimate historic human settlement in the wider landscape. The absence of villages in these colonial and post-colonial maps over time suggests a continuous disregard for village territories, and this tendency is also present in global maps.

### Global maps alone weaken conservation

Global maps such as the Intact Forest Landscape, which guide forestry concession management in Gabon (Forestry Stewardship Council 2020), cannot capture the historical and cultural aspects of a landscape, elements which are critical for local communities. Furthermore, they reproduce western ideas of forest conservation and landuse, displacing local perspectives (Savilaakso et al. 2023) and may risk promoting a false “terra nullius” idea of customary lands (Geisler 2012). In Massaha, the mapping of their biocultural territory enabled a better understanding of the area size, boundaries, and the identification of sites of specific interest, demonstrating the occupation of the forest by their ancestral communities prior to colonial times. It also enabled the community to launch the process of advocacy (Evine-Binet 2023) (see “Study site: Massaha and the Ogooué-Ivindo” and “Methods: Colonial maps” sections) to secure their land and its biocultural diversity and in doing so, strengthen the technical capacities and resilience of community members. In addition to identifying sites of interest, mapping enabled the identification of areas impacted by new industrial and illegal exploitation, better planning of community patrols, and it allowed the community to connect to their sites and their history (Evine-Binet 2023).

Concerning IFLs, this map suggests that industrial disturbances have not greatly impacted Massaha’s ancestral territory; yet, most of it has been logged. The most recent IFL data are from 2020, and updating it with GFW’s Integrated Deforestation Alerts would provide a more accurate picture. But still, satellite alerts failed to capture all logging recorded by community members during strategic patrols; relying on satellite data alone would almost double the estimated area of unlogged forest. A study of 50 sites across Central Africa found that GFW alerts generally provided accurate estimations of logging, though noted that “users should be aware that the reliability of estimations is relatively low in areas with few disturbances” (Welsink et al. 2023). However, their comparison was related to more accurate satellite data, not community mapping: more studies such as ours, across a variety of sites and contexts, would be insightful. In our study, locally collected ground data also provided leverage for forest defence (via government lobbying) in a way that satellite data were unable to, and revealed illegal practices unobservable by satellites. Furthermore, such data are important for understanding how logging impacts biodiversity like large mammals (Zwerts et al. 2024), and large trees (Sullivan et al. 2022), and also more subtle species like insects and small arboreal mammals (the impacts on which are just beginning to be understood). Bioacoustic monitoring conducted in Massaha’s forest showed that their unlogged forest was ‘louder’ at dawn and dusk than nearby logged forest (Yoh et al. 2024). With this study, we show that “who counts” as well as who does the counting (in this case, mapping) is vitally important to ensuring that conservation and natural resource maps accurately represent community territories; this is especially important when the counting, done by distant organisations, can invisibilise the interests of vulnerable groups (Rocheleau 1995).

Finally, in comparison with the colonial, post-colonial, and global landuse maps, Massaha’s community biocultural maps show that many parts of the IFL have been historically inhabited. These historical territories, such as that of Massaha, while not visible on an IFL map, constitute a biocultural territory which this community desires to conserve, and care for, sparing it from industrial logging. Without Massaha’s maps which demonstrate their long-term settlement and defence from illegal logging, this part of the forest appears on maps as empty and possibly without rightsholders. By contrast, Massaha’s maps clearly show the extent of their territory and the possibility to make a territorial claim, removing it from the logging concession and enabling their conservation of it. The Massaha community's land claims prompted a national debate on the legal recognition and governance of such “territories of life” (sensu Borrini-Feyerabend 2024; ICCA Consortium 2021) as community conserved areas, which at the time of submission are being integrated into ongoing revisions to the Forestry Code.

### The danger of “big data” maps and the scaling of environmental knowledge

The scaling of maps can obscure local issues, in particular property rights, something that has been highlighted in previous studies from the early days of remote sensing (e.g. Rocheleau 1995). “Counter maps”, or those maps made by local communities to contest institutional maps, also emerged in this context (Peluso 1995; Robbins 2003). However, “big data” are on the rise in conservation efforts to facilitate the timely and automated collection and processing of information (Runting et al. 2020) and particularly in the case of real-time monitoring, global data platforms, and citizen science (Ayoola et al. 2024). As new conservation programmes promoted by institutions such as the World Bank foresee a “data-driven pathway for global conservation” (Dasgputa et al. 2025), this paper intervenes in conversations about the scaling of environmental knowledge in this emerging technological landscape filled with "big data". The issues of global maps versus local interests become exacerbated with maps generated by “big data”. Wyborn and Evans (2021) “argue that global maps constitute a particular, and problematic, form of global knowledge that erases local context and difference.”

"Big data" are often generated via artificial intelligence (AI). For example, AI is key to Global Forest Watch’s Integrated Deforestation Alerts which, in the case of Massaha, did not accurately reflect deforestation on the ground. Such data processes, building on largely existing data, will potentially exacerbate local inequalities related to land tenure, as seen in this study. They can also give the misleading impression that “enough” data already exists and can be accessed by anyone, anywhere, potentially minimising the importance of locally-grounded, community-produced data; in other words, “AI-fueled techno-optimism distracting from other actions” (Sandbrook 2025).

### Community-researcher collaborative mapping

Collaborations between researchers and local people can lead to shared perspectives and so deepen the understanding of the environment and, in this case, making the interpretation of maps more accurate (Davy et al. 2021). Our mapping approach used a participatory GIS created for the Gabonese context, but similar and more widespread technologies exist, such as Mapeo and Sapelli (Tarrant et al. 2023). Colonial, post-colonial and global landuse maps, even when combined, cannot replace local knowledge about settlement, sacred areas, and illegal logging. Regardless of the tools used, supporting Indigenous Peoples and local communities to map their territories is a critical step in recognising their contributions to biocultural landscapes (Zanjani et al. 2023). While many global analyses discuss the importance of this step, few show how to do it or confront global maps with local ones and help redefine how conservation is implemented at the local scale.

### Community maps revitalise customary power

At the local scale of a village territory, such as Massaha, colonial maps do not document villages consistently or demonstrate their movement over time. The Massaha village maps show an area that has been lived in over time, with former village sites and sacred areas dating back to at least the 1880s. Still, caution should be used when examining seemingly “empty” areas even in Massaha’s maps: the current version shows ancestral villages as points, whereas in reality they are areas of past homes, fields, and current hunting territories that can extend several kilometres (Walters et al. 2015). Oral histories, which are valuable historical records (Vansina 1985; Okoro 2008), are able to “address deep biases or silences in the historical world” (Sloan and Cave 2022), and so provide a counter point to the colonial maps (Grenand et al. 2017), filling in a forest, with important cultural sites, a Kota biocultural view of a territory.

The community of Massaha used maps to show their long-term occupation of their ancestral forest and support their claim to save it from logging. Maps were not their only evidence; Massaha also demonstrated their commitment to conservation by collaborating on research projects, all of which show their territory’s high biodiversity (Beirne et al. 2019; Nuñez et al. 2019; Froese et al. 2022; 2023). These maps and other evidence were supplied by Massaha to rights-based conservation organisations (e.g., ICCA Consortium, Rainforest Foundation UK, Forest Peoples Programme) and shared in the media to amplify their voice and encourage the government to listen to them.

**Fig. 6:**
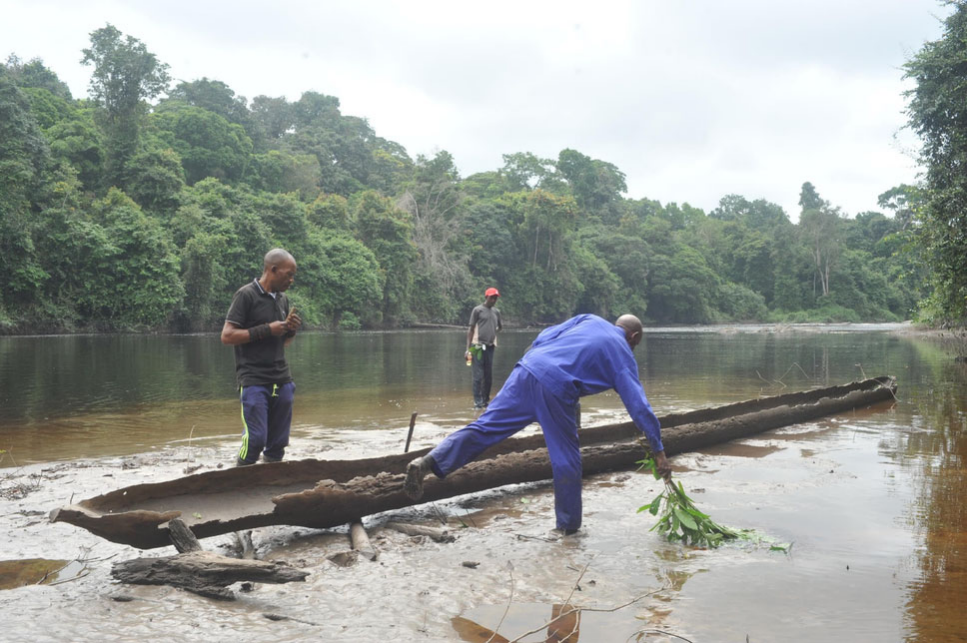
Co-authors Fulbert Makala, Modeste Ndongoabendje, and Dieu-Donné Ngouba perform a ceremony with Pendo (the name of a mystic snake that lays its eggs at the end of the rainbow), one of three remaining sacred canoes known as *boualôo* that Massaha exhumed after decades of being buried in the riverside. Photo by Graden Z.L. Froese, 2024. See Supplementary Information

Massaha’s mapping led to the revitalisation of cultural practices. In the community’s first letter to the government, they wrote of the *boualôo*, sacred, centennial canoes used in a customary form of fishing called *etoubili* (Fig. 5)*. Etoubili* had last been practised in 1980s, the three known remaining *boualôo* buried in riverside mud (Fig. 6). After logging had started to destroy ancestral villages and Massaha’s claim was still not being respected by governmental authorities, the community performed ceremonies, exhuming the *boualôo* to ask their spirits to protect and strengthen their struggle for forest rights (see Supplementary Information).

## Conclusion

In this paper, we show that global conservation mapping must account for biocultural mapping and other forms of local knowledge if they are to be effective in guiding conservation decisions. We call for attention to global mapping efforts and encourage them to work actively with local communities, especially when their maps and data-driven methods impact tenure, culture, and livelihoods. We move beyond the global mapping of forests and provide evidence of how bottom-up mapping by and with local communities provides a grounded view of the social history and conservation of these forests, which can lead to recognition of the long-term occupation of biocultural territories that communities wish to conserve. In doing so, community mapping instils an agency for achieving conservation action. The promotion and defence of community use and management rights against certain government decisions or actions by global corporations is a key result of local mobilisation (Villamayor-Tomas and García-López 2018) that is sometimes wrongly attributed to global mapping platforms (Schneider and Olman 2020). This critical case study is likely representative of other communities in Central Africa and elsewhere who are actively involved in protecting their resources. Laws and policies (Reyes-García et al. 2022) should be implemented to support them and further community territorial biocultural conservation efforts.

## Supplementary Information

Below is the link to the electronic supplementary material.

Supplementary file1 (PDF 104 KB)

## Data Availability

The data that support the findings of this study are available upon reasonable request and if consented by the community of Massaha through an FPIC process.

## Footnotes

1
https://data.globalforestwatch.org/datasets/gfw::integrated-deforestation-alerts/about.

2 https://www.rainforestfoundationuk.org/wp-content/uploads/2022/07/massahaletterfinal.pdf.

## References

[CR1] Areendran, G., M. Sahana, K. Raj, R. Kumar, A. Sivadas, A. Kumar, S. Deb, V. D. Gupta, et al. 2020. A Systematic Review on High Conservation Value Assessment (HCVs): Challenges and Framework for Future Research on Conservation Strategy. *Science of the Total Environment* 709: 135425. 10.1016/j.scitotenv.2019.135425. 31884271 10.1016/j.scitotenv.2019.135425

[CR2] Ayoola, V. B., I. P. Idoko, S. O. Eromonsei, O. Afolabi, A. R. Apampa, and O. S. Oyebanji. 2024. The Role of Big Data and AI in Enhancing Biodiversity Conservation and Resource Management in the USA. *World Journal of Advanced Research and Reviews* 23: 1851–1873. 10.30574/wjarr.2024.23.2.2350.

[CR3] Beirne, C., A. C. Meier, A. E. Mbele, G. M. Menie, G. Froese, J. Okouyi, and J. R. Poulsen. 2019. Participatory Monitoring Reveals Village-Centered Gradients of Mammalian Defaunation in Central Africa. *Biological Conservation* 233: 228–238. 10.1016/j.biocon.2019.02.035.

[CR4] Borrini-Feyerabend, G. 2024. *Territories of Life: Exploring Vitality of Governance for Conserved and Protected Areas*. ICCA Consortium. 10.70841/VY54762.

[CR5] Braucher, R., R. Oslisly, I. Mesfin, and P. P. Ntoutoume. 2022. In Situ-Produced 10Be and 26Al Indirect Dating of Elarmékora Earlier Stone Age Artefacts: First Attempt in a Savannah Forest Mosaic in the Middle Ogooué Valley, Gabon. *Philosophical Transactions of the Royal Society b: Biological Sciences* 377: 20200482. 10.1098/rstb.2020.0482. 10.1098/rstb.2020.0482 PMC8899616 35249387

[CR6] Brouwer, M. 2021. Certification Is the Future of the Industry. In *Central African Forests Forever*.

[CR7] Brunschwig, H. 1972. *Brazza Explorateur: Les Traités Makoko (1880–1882)*. Mouton & Co.

[CR8] Cinnamon, J. 2003. Narrating Equatorial African Landscapes: Conservation, Histories, and Endangered Forests in Northern Gabon. *Journal of Colonialism and Colonial History* 4.

[CR9] Conseil national climat Gabonais. 2020. *Gabon National Results Report: Results-Based Payments under the Central African Forest Initiative – Gabon Partnership*. Conseil national climat Gabonais.

[CR10] Coquery-Vidrovitch, C. 1972. *Le Congo Au Temps Des Grandes Compagnies Concessionnaires 1898–1930*. Mouton & Co.

[CR11] Country, B., S. Wright, S. Suchet-Pearson, K. Lloyd, L. Burarrwanga, R. Ganambarr, M. Ganambarr-Stubbs, B. Ganambarr, et al. 2016. Co-Becoming Bawaka: Towards a Relational Understanding of Place/Space. *Progress in Human Geography* 40: 455–475. 10.1177/0309132515589437.

[CR12] Dasgupta, S., B. Blankespoor, and D. Wheeler. (n.d.). The 30x30 Biodiversity Challenge: A Data-Driven Pathway for Global Conservation. World Bank Blogs. https://blogs.worldbank.org/en/opendata/the-30x30-biodiversity-challenge--a-data-driven-pathway-for-glob. Accessed 30 Nov 2025.

[CR13] Davy, D., P. Grenand, and G. Odonne. 2021. Ethnoecology of Landscape Uses and Interpretations. In *Methods in Historical Ecology: Insights from Amazonia*, ed. G. Odonne and J.-F. Molino. Milton Park: Routledge.

[CR14] Deschamps, H. 1962. *Traditions Orales et Archives Au Gabon*. Berger-Levrault.

[CR15] Doumenge, C., F. Palla, and G-L. Itsoua Madzous. 2021. *Aires Protégées d’Afrique Centrale – État 2020*. OFAC-COMIFAC, Yaoundé, Cameroun & UICN. https://www.observatoire-comifac.net/publications/edap/2020.

[CR16] Ellis, E. C., N. Gauthier, K. K. Goldewijk, R. Bliege Bird, N. Boivin, S. Díaz, D. Q. Fuller, J. L. Gill, et al. 2021. People Have Shaped Most of Terrestrial Nature for at Least 12000 Years. *Proceedings of the National Academy of Sciences* 118: e2023483118. 10.1073/pnas.2023483118. 10.1073/pnas.2023483118 PMC8092386 33875599

[CR17] Environmental Systems Research Institute. 2023. *ESRI*.

[CR18] Evine-Binet, B. 2023. Territory of Life: The Story of Ibola Dja Bana Da Massaha, the Reserve of All Massaha Children. *Langscape* 12: 34–39.

[CR19] Fairhead, J., and M. Leach. 1996. *Misreading the African Landscape: Society and Ecology in a Forest Savanna Mosaic*. Cambridge: Cambridge University Press.

[CR20] Flyvbjerg, B. 2011. Case Study. In *The SAGE Handbook of Qualitative Research*, ed. N. K. Denzin and Y. S. Lincoln. Thousand Oaks: SAGE.

[CR21] Forest, M., D. M. Kobei, S. L. Luari, D. Carroll, S. Gougsa, V. Pratt, N. Redvers, et al. 2025. ‘Removing an Ogiek from the Forest Is like Removing a Fish from Water’: A Qualitative Examination on Ogiek Community Impacts from Forced Land Eviction for Conservation. *PLOS Global Public Health* 5: e0004460. 10.1371/journal.pgph.0004460. 40560883 10.1371/journal.pgph.0004460 PMC12192252

[CR22] Forestry Stewardship Council. 2020. *The FSC National Forest Stewardship Standard of The Gabonese Republic*. FSC-STD-GAB-02–2020 EN. Forestry Stewardship Council. https://connect.fsc.org/document-centre/documents/resource/273.

[CR23] Froese, G. Z. L., A. E. Mbélé, C. Beirne, L. Atsame, C. Bayossa, B. Bazza, M. Bidzime Nkoulou, S. Dzime N’noh, et al. 2022. Coupling Paraecology and Hunter GPS Self-follows to Quantify Village Bushmeat Hunting Dynamics across the Landscape Scale. *African Journal of Ecology* 60: 229–249. 10.1111/aje.12956.

[CR24] Froese, G. Z. L., A. E. Mbélé, C. Beirne, B. Bazza, S. Dzime N’noh, J. Ebeba, J. Edzidzie, S. Ekazama Koto, et al. 2023. Fluid Hunter Motivation in Central Africa: Effects on Behaviour, Bushmeat and Income. *People and Nature* 5: 1480–1496. 10.1002/pan3.10502.

[CR25] Garnett, S. T., N. D. Burgess, J. E. Fa, A. Fernández-Llamazares, Z. Molnár, C. J. Robinson, J. E. Watson, K. K. Zander, et al. 2018. A Spatial Overview of the Global Importance of Indigenous Lands for Conservation. *Nature Sustainability* 1: 369–374. 10.1038/s41893-018-0100-6.

[CR26] Gavin, M. C., J. McCarter, F. Berkes, A. T. Mead, E. J. Sterling, R. Tang, and N. J. Turner. 2018. Effective Biodiversity Conservation Requires Dynamic, Pluralistic, Partnership-Based Approaches. *Sustainability* 10: 1846. 10.3390/su10061846.

[CR27] Geisler, C. 2012. New Terra Nullius Narratives and the Gentrification of Africa’s “Empty Lands.” *Journal of World-Systems Research *XVIII(1):15–29. 10.5195/jwsr.2012.484.

[CR28] Grenand, P., F. Grenand, P. Joubert, and D. Davy. 2017. Pour une histoire de la cartographie des territoires teko et wayãpi (Commune de Camopi, Guyane française). *Revue D’ethnoécologie,* 11: 11. 10.4000/ethnoecologie.3007.

[CR29] Hackeloeer, A., K. Klasing, J. M. Krisp, and L. Meng. 2014. Georeferencing: A Review of Methods and Applications. *Annals of GIS* 20: 61–69. 10.1080/19475683.2013.868826.

[CR30] Harley, J. B. 1988. Maps, Knowledge and Power. In *The Iconography of Landscape*, ed. D. Cosgrpve and S. Daniels. Cambridge: Cambridge University Press.

[CR31] Harris, L. M., and H. D. Hazen. 2005. Power of Maps: (Counter) Mapping for Conservation. *ACME: an International Journal for Critical Geographies* 4: 99–130.

[CR32] Haurez, B., K. Daïnou, C. Vermeulen, F. Kleinschroth, F. Mortier, S. Gourlet-Fleury, J. L. Doucet, et al. 2017. A Look at Intact Forest Landscapes (IFLs) and Their Relevance in Central African Forest Policy. *Forest Policy and Economics* 80: 192–199. 10.1016/j.forpol.2017.03.021.

[CR33] Hubau, W., S. L. Lewis, O. L. Phillips, K. Affum-Baffoe, H. Beeckman, A. Cuní-Sanchez, A. K. Daniels, C. E. Ewango, et al. 2020. Asynchronous Carbon Sink Saturation in African and Amazonian Tropical Forests. *Nature* 579: 7797. 10.1038/s41586-020-2035-0. 10.1038/s41586-020-2035-0 PMC7617213 32132693

[CR34] ICCA Consortium. 2021. *Territories of Life: 2021 Report*. ICCA Consortium. https://report.territoriesoflife.org/executive-summary/.

[CR35] Kleinschroth, F., N. Laporte, W. F. Laurance, S. J. Goetz, and J. Ghazoul. 2019. Road Expansion and Persistence in Forests of the Congo Basin. *Nature Sustainability* 2: 628. 10.1038/s41893-019-0310-6.

[CR36] Legault, D., and L. Cochrane. 2021. Forests to the Foreigners: Large-Scale Land Acquisitions in Gabon. *Land* 10: 420. 10.3390/land10040420.

[CR37] Liboiron, M., A. Zahara, and I. Schoot. 2018. Community Peer Review: A Method to Bring Consent and Self-Determination into the Sciences. *Preprints*, ahead of print, June 7. 10.20944/preprints201806.0104.v1.

[CR38] Mabika Ognandzi, H. 2019. *Walter Munz: Dans La Suite d’Albert Schweitzer à Lambaréné: Une Biographie*. Favre: Biographie.

[CR39] Matias, G., F. Cagnacci, and L. M. Rosalino. 2024. FSC Forest Certification Effects on Biodiversity: A Global Review and Meta-Analysis. *Science of the Total Environment* 908: 168296. 10.1016/j.scitotenv.2023.168296. 37926251 10.1016/j.scitotenv.2023.168296

[CR40] Métégué N’Nah, N. 2006. *Histoire Du Gabon: Des Origines à l’aube Du XXIe Siècle*. Harmattan: Etudes Africaines.

[CR41] Molnár, Z., Y. Aumeeruddy-Thomas, D. Babai, S. Díaz, S. T. Garnett, R. Hill, P. Bates, E. S. Brondízio, et al. 2023. Towards Richer Knowledge Partnerships Between Ecology and Ethnoecology. *Trends in Ecology & Evolution* 39: 109–115. 10.1016/j.tree.2023.10.010. 37981565 10.1016/j.tree.2023.10.010

[CR42] Morin-Rivat, J., A. Fayolle, C. Favier, L. Bremond, S. Gourlet-Fleury, N. Bayol, P. Lejeune, H. Beeckman, et al. 2017. Present-Day Central African Forest Is a Legacy of the 19th Century Human History. *eLife* 6: e20343. 10.7554/eLife.20343. 28093097 10.7554/eLife.20343 PMC5241113

[CR43] M’sit, N., A. Marshall, K. F. Beazley, J. Hum, S. Joudry, A. Papadopoulos, S. Pictou, J. Rabesca, et al. 2021. ‘Awakening the Sleeping Giant’: Re-Indigenization Principles for Transforming Biodiversity Conservation in Canada and Beyond. *FACETS* 6: 839–869. 10.1139/facets-2020-0083.

[CR44] Myers, N., R. A. MIttermeier, C. G. Mittermeier, G. A. B. da Fonseca, and J. Kent. 2000. Biodiversity Hotspots for Conservation Priorities. *Nature* 403: 853–858. 10706275 10.1038/35002501

[CR45] Nuñez, C. L., G. Froese, A. C. Meier, C. Beirne, J. Depenthal, S. Kim, A. E. Mbélé, A. Nordseth, et al. 2019. Stronger Together: Comparing and Integrating Camera Trap, Visual, and Dung Survey Data in Tropical Forest Communities. *Ecosphere* 10: e02965. 10.1002/ecs2.2965.

[CR46] Okoro, J. A. 2008. Reflections on the Oral Traditions of the Nterapo of the Salaga Area. *History in Africa* 35: 375–400. 10.1353/hia.0.0000.

[CR47] Olive, B. 2019. *Cartes et Plans Isolés: Série PL (Répertoire Méthodique)*. Archives Nationales d’Outre-Mer.

[CR48] Olivero, J., J. E. Fa, M. A. Farfán, J. Lewis, B. Hewlett, T. Breuer, G. M. Carpaneto, M. Fernández, et al. 2016. Distribution and Numbers of Pygmies in Central African Forests. *PLoS ONE* 11: e0144499. 10.1371/journal.pone.0144499. 26735953 10.1371/journal.pone.0144499 PMC4711706

[CR49] Oslisly, R. 2001. The History of Human Settlement in the Middle Ogooué Valley (Gabon): Implications for the Environment. In *African Rain Forest Ecology and Conservation: An Interdisciplinary Perspective*, ed. B. Weber, J. T. W. Lee, A. Vedder, and L. Naughton-Trevis. Yale University Press.

[CR50] Palsky, G. 2013. Cartographie participative, cartographie indisciplinée. *L’information Géographique (Paris)* 77: 10–25. 10.3917/lig.774.0010.

[CR51] Peluso, N. L. 1995. Whose Woods Are These? Counter-Mapping Forest Territories in Kalimantan, Indonesia. *Antipode* 27: 383–406. 10.1111/j.1467-8330.1995.tb00286.x.

[CR52] Perrois, L. 1970. Chronique Du Pays Du Kota (Gabon). *Cahiers D’orstom, Série Sciences Humaines* VII: 16–110.

[CR53] Potapov, P., M. C. Hansen, L. Laestadius, S. Turubanova, A. Yaroshenko, C. Thies, W. Smith, I. Zhuravleva, et al. 2017. The Last Frontiers of Wilderness: Tracking Loss of Intact Forest Landscapes from 2000 to 2013. *Science Advances* 3: e1600821. 10.1126/sciadv.1600821. 28097216 10.1126/sciadv.1600821 PMC5235335

[CR54] Pourtier, R. 1989. *Le Gabon Tome 2: Etat et Développement*. Harmattan.

[CR55] Quammen, D. 2003. Saving Africa’s Eden. *National Geographic* 203: 50–74.

[CR56] Reyes-García, V., Á. Fernández-Llamazares, Y. Aumeeruddy-Thomas, P. Benyei, R. W. Bussmann, S. K. Diamond, D. García-del-Amo, S. Guadilla-Sáez, et al. 2022. Recognizing Indigenous Peoples’ and Local Communities’ Rights and Agency in the Post-2020 Biodiversity Agenda. *Ambio* 51: 84–92. 10.1007/s13280-021-01561-7. 34008095 10.1007/s13280-021-01561-7 PMC8651947

[CR57] Robbins, P. 2003. Beyond Ground Truth: GIS and the Environmental Knowledge of Herders, Professional Foresters, and Other Traditional Communities. *Human Ecology* 31: 233–253.

[CR58] Rocheleau, D. 1995. Maps, Numbers, Text, and Context: Mixing Methods in Feminist Political Ecology. *The Professional Geographer* 47: 458–466. 10.1111/j.0033-0124.1995.458_h.x.

[CR59] Rossinelli, F. 2022. *Géographie et impérialisme: De la Suisse au Congo entre exploration géographique et conquête coloniale*. Histoire: Éditions Alphil-Presses universitaires suisses.

[CR60] Runting, R. K., S. Phinn, Z. Xie, O. Venter, and J. E. M. Watson. 2020. Opportunities for Big Data in Conservation and Sustainability. *Nature Communications* 11: 2003. 10.1038/s41467-020-15870-0. 10.1038/s41467-020-15870-0 PMC7181767 32332744

[CR61] Sandbrook, C. 2025. Beyond the Hype: Navigating the Conservation Implications of Artificial Intelligence. *Conservation Letters* 18: e13076. 10.1111/conl.13076.

[CR62] Sautter, G. 1966. *De l’Atlantique Au Fleuve Congo, Une Géographie Du Sous-Peuplement. République Du Congo, République Du Gabon*. Editions du Centre National de la Recherche Scientifique. Paris, France.

[CR63] Savilaakso, S., N. Lausberg, P. O. Waeber, O. Hillgén, A. Isotalo, F. Kleinschroth, I. N. Djenontin, N. B. Lefeuvre, et al. 2023. Whose Perspective Counts? A Critical Look at Definitions of Terms Used for Natural and near-Natural Forests. *One Earth* 6: 1477–1493. 10.1016/j.oneear.2023.10.003.

[CR64] Schneider, B., and L. Olman. 2020. The Geopolitics of Environmental Global Mapping Services: An Analysis of Global Forest Watch. In *A Research Agenda for Environmental Geopolitics*. Edward Elgar Publishing. https://www.elgaronline.com/edcollchap/edcoll/9781788971232/9781788971232.00010.xml.

[CR65] Sloan, S. M., and M. Cave, eds. 2022. *Oral History and the Environment: Global Perspectives on Climate, Connection, and Catastrophe*. Oxford University Press.

[CR66] Soret, M. 1973. *Les Téké de l’est. Essai Sur l’adaptation d’une Population à Son Milieu*. Université de Lille.

[CR67] Sullivan, M. K., P. A. M. Biessiemou, R. Niangadouma, K. Abernethy, S. A. Queenborough, and L. Comita. 2022. A Decade of Diversity and Forest Structure: Post-Logging Patterns across Life Stages in an Afrotropical Forest. *Forest Ecology and Management* 513: 120169. 10.1016/j.foreco.2022.120169.

[CR68] Sze, J. S., L. Roman Carrasco, D. Childs, and D. P. Edwards. 2022. Reduced Deforestation and Degradation in Indigenous Lands Pan-Tropically. *Nature Sustainability* 5: 123–130. 10.1038/s41893-021-00815-2.

[CR69] Sagang, T., L. Bienfaiteur, S. Favrichon, R. Dalagnol, V. Medjibe, F. Manfoumbi, C. Obame, F. Wagner, S. George-Chacon, et al. 2024. Unveiling Spatial Variations of High Forest Live Biomass Carbon Stocks of Gabon Using Advanced Remote Sensing Techniques. *Environmental Research Letters* 19: 074038. 10.1088/1748-9326/ad5572.

[CR70] Tarrant, M., M. Moreu, H. M. B. Gibbs, M. Haklay, J. Lewis, M. Laws, A. Skarlatidou, F. Moustard, et al. 2023. Sapelli. In *Evaluating Participatory Mapping Software*, ed. C. M. Burnett. Bern: Springer. 10.1007/978-3-031-19594-5_5.

[CR71] Tsanga, R., S. Assembé-Mvondo, G. Lescuyer, C. Vermeulen, D. A. Wardell, M. A. Kalenga, L. Boutinot, P. R. Oyono, et al. 2022. Les Droits Des Populations Locales et Autochtones à l’épreuve Des Politiques Forestières et de Conservation. In Les forêts du Bassin du Congo : état des Forêts 2021, ed. R. Ebaá Atyi, F. Hiol Hiol, G. Lescuyer, et al. CIFOR. 10.17528/cifor/008565.

[CR72] Villamayor-Tomas, S., and G. García-López. 2018. Social Movements as Key Actors in Governing the Commons: Evidence from Community-Based Resource Management Cases across the World. *Global Environmental Change* 53: 114–126. 10.1016/j.gloenvcha.2018.09.005.

[CR73] Walters, G., O. Hymas, S. Touladjan, and K. Ndong. 2025. Revealing the Social Histories of Ancient Savannas and Intact Forests Using a Historical Ecology Approach in Central Africa. In *The Field Guide to Mixing Social and Biophyiscal Methods in Environmental Research*, ed. R. Lave and S. Lane. Open Book Publishing. 10.11647/OBP.0418.

[CR74] Walters, G., J. Schleicher, O. Hymas, and L. Coad. 2015. Evolving Hunting Practices in Gabon: Lessons for Community-Based Conservation Interventions. *Ecology and Society* 20. 10.5751/ES-08047-200431.

[CR75] Walters, G., and D. Andrew Wardell. 2024. The Rise and Fall of Protected Areas in Central Africa: A Historical Perspective. In *Power Dynamics and Biodiversity Crisis in African Forests*, ed. S. Ongolo and M. Krott. Routledge.

[CR76] Watson, J. E. M., T. Evans, O. Venter, B. Williams, A. Tulloch, C. Stewart, I. Thompson, J. C. Ray, et al. 2018. The Exceptional Value of Intact Forest Ecosystems. *Nature Ecology & Evolution* 2: 599–610. 10.1038/s41559-018-0490-x. 29483681 10.1038/s41559-018-0490-x

[CR77] Welsink, A.-J., J. Reiche, V. de Sy, S. Carter, B. Slagter, D. R. Suarez, B. Batros, M. Peña-Claros, et al. 2023. Towards the Use of Satellite-Based Tropical Forest Disturbance Alerts to Assess Selective Logging Intensities. *Environmental Research Letters* 18: 054023. 10.1088/1748-9326/acd018.

[CR78] Wyborn, C., and M. C. Evans. 2021. Conservation Needs to Break Free from Global Priority Mapping. *Nature Ecology & Evolution* 5: 10. 10.1038/s41559-021-01540-x. 34426678 10.1038/s41559-021-01540-x

[CR79] Yoh, N., W. Mbamy, B. L. Gottesman, G. Z. Froese, T. Satchivi, M. O. Ebanega, L. Carlson, S. E. Koto, et al. 2024. Impacts of Logging, Hunting, and Conservation on Vocalizing Biodiversity in Gabon. *Biological Conservation* 296: 110726. 10.1016/j.biocon.2024.110726.

[CR80] Zanjani, L. V., H. Govan, H. C. Jonas, T. Karfakis, D. M. Mwamidi, J. Stewart, G. Walters, P. Dominguez, et al. 2023. Territories of Life as Key to Global Environmental Sustainability. *Current Opinion in Environmental Sustainability* 63: 101298. 10.1016/j.cosust.2023.101298.

[CR81] Vansina, J. M. 1985. *Oral Tradition as History*. Univ of Wisconsin Press.

[CR82] Zwerts, J. A., E. H. M. Sterck, P. A. Verweij, F. Maisels, J. van der Waarde, E. A. Geelen, G. B. Tchoumba, H. F. Donfouet Zebaze, et al. 2024. FSC-Certified Forest Management Benefits Large Mammals Compared to Non-FSC. *Nature* 628: 563–568. 10.1038/s41586-024-07257-8. 38600379 10.1038/s41586-024-07257-8 PMC11023928

