# Supplementary material for: Community biocultural mapping reveals historical occupation and enables defense of African rainforests
Source: Ambio. 2026 Feb 20;55(8):1830–44. doi: 10.1007/s13280-025-02334-2 (PMC13319623; doi:10.1007/s13280-025-02334-2)
Supplement: Supplementary file 1 — Supplementary file1 (PDF 104 KB) [file 13280_2025_2334_MOESM1_ESM.pdf]

Ambio

Supplementary Information

Community biocultural mapping reveals historical occupation and enables defense of African rainforests

See next page for the story of etoubili & boalôo...

Etoubili and boalôo

[The text below was orated by Serge Ekazama Kota on 11/09/24 in Massaha, transcribed as translated from French by Graden Zane Lambert Froese. Translator notes are in square brackets.]

Etoubili was a type of fishing practiced by our grandparents. After our grandparents died they transmitted the knowledge to our papas. And my papas explained to me that etoubili is different than all other types of fishing. It was a type of fishing only practiced during the long holiday. And that fishing was by clan, it wasn't everybody that had access. There was ngonguey, there were other clans, we are not many, there must be three or four [clans]. Before etoubili we took our time, we went first to search for a liana we call anonguey [scientific name unknown]. When we found a lot of anonguey we would mark the area and look for where to establish a camp. The camps where the men would stay. This was in the dry season. Before entering the camp, men would first go cut the anonguey and bring it to a special place to clean it, only men, and then weave the nets, they had to be at least six or ten meters. After the weaving everything was ready. Then we'd tell the men and women to go to the camp. But before entering the camp there were what we call sacrifices. A traditional dance the day we entered camp. And a young man who we would choose to cut a small liana, that would make the fish be caught in abundance. We'd treat that young man like we treat a child before a circumcision in August. He would stay apart from the others, be treated differently. His food, an old mama would have to prepare his food. And certain things in the camp were prohibited. If you stayed two or three months, you'd sleep with your wife without sexual intercourse. Those are the prohibitions there. And the nganga, he who initiates, he who must give all, who masters, who has the monopoly of [spiritual] power, who was in relation with spirits, he was also apart from the others, not far from the young boy, who he would watch over. The young boy couldn't eat food from a women who was on her period. All that. For the fishing to be in abundance, lots of fish, there needed to be a traditional pirouge that we call the boalôo. The boalôo we carved from the bilinga tree [*Nauclea diderrichii*, Near Threatened on the IUCN Red List]. To carve a boalôo first you'd have to find a bilinga tree, and then fell it. And it wasn't just anyone that would fell it. You have to do a sacrifice at the foot of the bilinga tree. And those who were chosen to fell the tree, they'd have to do it with only one stroke a day. A stroke a day, a stroke a day, until the tree fell. And we carved the whole tree. We dug a long hole in the middle. The tree could be 14 meters, you carved the whole trunk. That's the pirogue we call the boalôo. And the boalôo is in contact with the genies. If you don't have the vampire, if you don't have the panther [*Panthera pardus*, Vulnerable on the IUCN Red List], if you're not in contact with the spirits, you couldn't be the master initiator of

etoubili. You have to be a person with mastery, with power, a sorcerer, when we speak about sorcerers we mean to have the vampire. But after he carved the boalôo we had to pull it to the water. And when we pulled it to the water we had to do a sacrifice. It was the nganga, the master initiator of etoubili, who did the sacrifice. It was the nganga who spoke with the boalôo. And it was the boalôo who permitted us to have lots of fish. And there was other traditional medicine we used, with the bones of grand-parents or great-grandparents, and it is those grand-parents that transform, because in our totems me I'm from the clan ngonguey, our totem is the caiman, the crocodile [*Mecistops cataphractus*, Criticially Endagnered on the IUCN Red List], there are people from our clan who transform into crocodiles, and it's them who when we put the net in the water during etoubili, they are around and they chase the fish into the net, and when we haul up the net, we have an abundance of fish. And those fish, sometimes we fill ten pirgoues. But we couldn't take every pirogue all at once, but depending on the number of people in the clan, we'd have to release at least two pirogues of fish back into the water. And that is why during the period of that type of fishing there is always fish. And our parents knew all that. And at the end of the fishing, the part we gave you was your right, even if you're the nephew or the grandson, if you brought your wife or father-in-law, if we had fish there was fish for you. So the nganga can't ask you to give him your part, he had his part and you had your part. And that's how we did that fishing. And that fishing permitted people to reinforce their links with our genies.

But after 1978, the last time that our parents practiced that fishing, after 1978 we lost our papas, our papas died, and our generation judged it useful to abandon that fishing because nobody from our generation could support the prohibitions that were practiced. So that's how since 1978 the pirgoues, the boalôo became buried in the mud. Given the importance of our request to the administration to secure our old villages and sacred sites, knowing that the boalôo was a sacred pirogue that possesses the power of the spirits, the presence of the logging company in our forest, that truly caused danger for our sacred sites. Even if the boalôo were underwater, but those who are linked to that fishing or those pirogues, every time before going [modern forms of] fishing, they still have to give a little sacrifice so that their fishing is abundant. And that's how in securing, in writing the administration to secure our old villages and sacred sites, we went to demonstrate that what we are doing is not a lie, we needed to exhume the three boalôo where they were found, from the mud. The names of those three boalôo, there are two that I master, there's Ngoubou, there's Pendo, and the other is the boalôo of ndongo. And we resurfaced them all to show the administration that what we are asking for is not a lie but the reality. Because if today we ruin and destroy our sacred sites, we will have lost many things to us. History, we'll have lost our history.

That's why I am here to tell you that it is really really important that etoubili, in relation to the boalôo, that is what our parents practiced, and that we ourselves dug up the boalôo, the boalôo they exist "belle et bien", and the boalôo bring us many things. The proof, this year, during the long holiday, we really had abundance, especially for those who went to the river to camp and fish. Because before the long holiday, in May and July, we went to the feet [of the trees], because where the boalôo are found are found the tree called the kévazingo [*Guibourtia tessmannii,* Endangered on the IUCN Red List], and the kévazingo don't grow on dry land but moreover beside water in swampy areas, and that which is curious is that where boalôo are found there is always the presence of a kévazingo. They are sacred sites. We went to Pendo and Ngoubou, to beg them, to ask them, that this year we could have lots and lots of fish in our river the Liboumba. And that's what happened and we saw the reality, the fruits of our labour, in asking, in begging the boalôo to free us the forest and river. And that's what was done, and the witness are in the village, and that's what I am telling you. Etoubili is a fishing that our parents practiced, and we as well near the end of their reign we also took part in that fishing, that we no longer practice that but there are still signs that remain, especially for the families linked with the fishing etoubili and the pirogues boalôo. We have a strong link with our traditional rites. I thank you.
